# Low T-cell subsets prior to development of virus-associated cancer in HIV-seronegative men who have sex with men

**DOI:** 10.1007/s10552-018-1090-4

**Published:** 2018-10-12

**Authors:** Anupriya Dutta, Hajime Uno, David R. Lorenz, Steven M. Wolinsky, Dana Gabuzda

**Affiliations:** 1 Department of Cancer Immunology and Virology, Dana Farber Cancer Institute, Center for Life Science 1010, 450 Brookline Avenue, Boston, MA 02215 USA; 2 Department of Medical Oncology, Dana-Farber Cancer Institute, Boston, MA 02215 USA; 3 Division of Infectious Diseases, Department of Medicine, Northwestern University Feinberg School of Medicine, Chicago, IL 60611 USA

**Keywords:** Cancer risk, Oncogenic viruses, HBV, CD4 T cells, CD8 T cells, Lymphopenia

## Abstract

Immunological parameters that influence susceptibility to virus-associated cancers in HIV-seronegative individuals are unclear. We conducted a case–control cohort study of immunological parameters associated with development of incident virus-associated cancers among 532 HIV-seronegative men who have sex with men (MSM) enrolled in the Multicenter AIDS Cohort Study (MACS) with median (IQR) 21 (8–26) years of follow-up. Thirty-two incident virus-associated cancers (anal cancer, non-Hodgkin lymphoma, liver cancer, other cancers with etiologies linked to human papillomavirus, Epstein–Barr virus, hepatitis B virus, or human herpesvirus-8) were identified among 3,408 HIV-seronegative men in the MACS during 1984–2010. Cases were matched for demographics, smoking, and follow-up to 500 controls without cancer. Mixed-effects and Cox regression models were used to examine associations between nadir or recent CD4, CD8, and white blood cell (WBC) counts or CD4:CD8 ratios and subsequent diagnosis of virus-associated cancers. Men with incident virus-associated cancers had lower CD4 and WBC counts over a 6-year window prior to diagnosis compared to men without cancer (*p* = 0.001 and 0.03, respectively). Low CD4 cell count and nadir, CD4 count-nadir differential, and CD4:CD8 ratio nadir were associated with increased 2-year risk of incident virus-associated cancers in models adjusted for demographics and smoking (hazard ratios 1.2–1.3 per 100 or 0.1 unit decrease, respectively; *p* < 0.01). Other associated factors included heavy smoking and past or current hepatitis B virus infection. These findings show that low CD4 cell counts, CD4 nadir, and CD4:CD8 cell ratios are independent predictors for subsequent risk of virus-associated cancers in HIV-seronegative MSM.

**Electronic supplementary material:**

The online version of this article (10.1007/s10552-018-1090-4) contains supplementary material, which is available to authorized users.

## Introduction

Viral infections are likely etiological agents in nearly one-fifth of cancer cases [1–3]. Virus-associated cancers with causes linked to oncogenic viruses include genital, anal, and oral cancers (HPV), non-Hodgkin and Hodgkin lymphomas (EBV), Kaposi sarcoma (HHV-8), and liver cancer (HBV). However, the oncogenic potential of viruses such as HPV, EBV, HHV-8, and HBV is challenging to ascertain due to the multifactorial nature of cancer development [3, 4]. Pinpointing viral origins of cancers is complicated by several factors, including prolonged viral latency before tumors arise, involvement of host and environmental co-factors in tumorigenesis, and host variability in natural immune mechanisms that protect against tumor development [1, 2].

The immune response to infection by oncogenic viruses is a key factor that helps to explain why only a fraction of these viral infections result in tumors [2, 5]. Immunosuppression and inflammation are co-factors for viral carcinogenesis [2, 6, 7], and cancers with viral etiologies occur more frequently in immunosuppressed populations, including HIV-infected and transplant patients [6, 8–14]. Primary immunodeficiencies such as idiopathic CD4+ lymphopenia (CD4 T-cell count < 300 cells/µl) and idiopathic neutropenia (neutrophil count < 200 cells/µl) are rare disorders that have been associated with elevated risk of virus-associated cancers [15]. Cytopenias can also occur in patients with cirrhosis [16, 17], while subclinical immune dysfunction has been associated with liver fibrosis [18]. Immunological parameters predictive for development of virus-associated cancers and their relationship to liver diseases in the general population are unclear.

The prognostic value of immunological factors in determining treatment responses underscores their importance for cancer immunotherapies [6, 19], and suggests that natural immune protection and immunosurveillance can influence cancer susceptibility. However, few studies have evaluated immunologic risk profiles associated with development of specific types of virus-associated cancers with etiologies linked to HPV, EBV, HBV, HCV, or HHV-8 in HIV-seronegative individuals [6]. Here, we conducted a longitudinal matched case–control study of HIV-seronegative men who have sex with men (MSM) in the MACS (Multicenter AIDS Cohort Study) to evaluate immunological predictors of virus-associated cancers.

## Methods

### Study cohort

This is a matched case–control study of men enrolled in the MACS, an ongoing natural history cohort study of men who report sex with men. The MACS was established in 1984, enrolling 6,972 HIV-infected and HIV-uninfected MSM over three recruitment waves [1984–1985 (*n* = 4,957), 1987–1991 (*n* = 665), 2001–2003 (*n* = 1,350)] across four study sites (Los Angeles, Chicago, Baltimore, and Pittsburgh). Behavioral, clinical, and laboratory data including HIV serology tests were collected at semi-annual visits as described [20, 21]. Institutional Review Boards at each study site approved the research, and all participants provided written informed consent.

Participants for this study were 532 HIV-seronegative men over age 18 followed between 1984 and 2010 with no pre-existing cancers and ≥ 2 visits with values for immunological parameters. Thirty-two cases with incident virus-associated cancers were identified through a database search of cancer diagnoses among all HIV-seronegative participants in the MACS (*n* = 3,408). For cohort assembly, an initial search identified 25 incident virus-associated cancers that were matched 1:20 to 500 controls without cancer for age at endpoint, race, smoking, and calendar period using the MatchIT package in R version 3.2.4. We later identified seven additional incident cancers and examined whether case–control matching was still balanced after inclusion of these cases; demographics and smoking were still matched (*p* > 0.20) (Table 1), so we included these additional cases to increase precision of the analysis. HIV-negative serostatus was confirmed at study visits within 2 years of cancer diagnosis for 29 cases and within 4–5 years for three cases. Because all cases remained HIV-seronegative during follow-up, HIV RNA viral load assays were performed for only three cases; all were below the limit of detection of the assay (< 50 copies per ml of plasma).

**Table 1:** Demographic and clinical characteristics by virus-associated cancer diagnosis

	All (*n* = 532)	Controls (*n* = 500)	Cases^a^ (*n* = 32)	*p*
Cumulative person years, median (IQR)	21 (8–26)	22 (8–26)	15 (8–21)	**0.003**
Age at entry visit, median (IQR)	35 (30–42)	35 (30–42)	42 (36–45)	< **0.001**
Age at endpoint, median (IQR)	54 (47–61)	54 (47–61)	57.5 (49–62.2)	0.22
Race				0.63
Non-black	423 (79.5)	396 (79.2)	27 (84.4)	
Black	109 (20.5)	104 (20.8)	5 (15.6)	
Heavy smoking^b^				0.37
No	424 (79.7)	401 (80.2)	23 (71.9)	
Yes	108 (20.3)	99 (19.8)	9 (28.1)	
Hepatitis C infection^c^	43 (8.1)	42 (8.4)	1 (3.1)	0.47
Hepatitis B infection^c^	22 (4.4)	15 (3.0)	7 (21.9)	< **0.001**
Sexually transmitted infection^c^				
Genital warts	168 (31.6)	160 (32)	8 (25)	0.53
Syphilis	74 (13.9)	68 (13.6)	6 (18.8)	0.58
CD4 count (cells/µl)^d^				0.32
< 200	0 (0)	0 (0)	0 (0)	
200–349	3 (0.6)	3 (0.6)	0 (0)	
350–499	17 (3.2)	14 (2.8)	3 (9.7)	
500–599	44 (8.3)	42 (8.4)	2 (6.5)	
≥ 600	467 (87.9)	441 (88.2)	26 (83.9)	
CD4 count (cells/µl)^d^, median (IQR)	943 (729–1,158)	947.5 (736–1,162)	858 (693–1,019)	0.15
CD4 nadir < 500 (cells/µl) ^c^	152 (28.6)	137 (27.4)	15 (46.9)	**0.03**
CD4 nadir (cells/µl)^c^, median (IQR)	614 (483–771)	620 (484–775)	512 (370–652)	**0.007**
CD8 count (cells/µl)^d^, median (IQR)	515 (381–693)	512 (383–685)	629 (378–759)	0.32
CD8 nadir < 250 (cells/µl)^c^	152 (28.6)	141 (28.2)	11 (34.4)	0.58
CD8 nadir (cells/µl)^c^, median (IQR)	323.5 (237–410)	326 (238–410)	304 (232–386)	0.69
CD4:CD8 ratio^d^, median (IQR)	1.84 (1.3–2.4)	1.85 (1.4–2.4)	1.62 (1.2–2.1)	0.08
CD4:CD8 ratio < 1^d^	44 (8.3)	39 (7.8)	5 (16.1)	0.20
CD4:CD8 ratio nadir^c^, median (IQR)	1.2 (0.93–1.5)	1.2 (0.94–1.6)	0.92 (0.72–1.4)	**0.001**
CD4:CD8 ratio nadir < 1^c^	164 (30.8)	146 (29.2)	18 (56.2)	**0.003**
White blood cell (WBC) count (cells/µl)^d^, median (IQR)	6,000 (5,000–7,500)	6,050 (5,000–7,500)	5,700 (5,000–7,400)	0.56
WBC nadir (cells/µl)^c^, median (IQR)	4,400 (3,800–5,300)	4,400 (3,800–5,300)	4,300 (3,400–5,225)	0.35
Cumulative sexual partners^e^ ≥ 10 partners	289 (54.3)	272 (54.4)	17 (54.8)	1.00
Median years to diagnosis from CD4 nadir			9.75 (6.0–14.8)	
Median years to diagnosis from CD8 nadir			8.5 (4.5–14.5)	
Median years to diagnosis from CD4:CD8 ratio nadir			8.17 (4.5–13.5)	
Median years to diagnosis from WBC nadir			7.25 (3.4–13.5)	

Data are *n* (%) unless otherwise indicated

Bold values indicate *p* < 0.05

^a^Composite measure of first virus-associated cancer diagnosis of Kaposi sarcoma (KS), non-Hodgkin lymphoma (NHL), Hodgkin lymphoma (HL), anal cancer, head and neck squamous cell carcinoma (HNSCC), or liver cancer

^b^0.5 packs/day for more than half the duration of follow-up

^c^Anytime following enrollment to study endpoint

^d^Nearest available value 1 year prior to endpoint

^e^Summarized over first three visits

### Data collection and risk factor classification

The MACS public dataset (P23 release) was translated to a local SQL database and used for the analyses. Immunological parameters and clinical characteristics associated with cancer risk factors were censored at 1 year prior to last follow-up visit for both cases and controls to account for a potential lag between clinical diagnosis of incident cancer and date of cancer diagnosis ascertained through registry linkage. CD4 and CD8 T-cell counts, white blood cell (WBC) counts, CD4/CD8 ratios, and Fibrosis-4 (FIB-4) score (a non-invasive index of liver fibrosis calculated from age, aspartate aminotransferase (AST), alanine aminotransferase (ALT), and platelet count; FIB-4 < 1.45 has a negative predictive value of 90% for advanced fibrosis) [22] were summarized as time-varying variables using values nearest to 1 year before last follow-up visit. Nadir values were the lowest value during follow-up. Time-updated smoking was defined as smoking on average more than half a pack per day within last 6 years of follow-up. Hepatitis C virus (HCV) infection status was determined by positive HCV antibody or RNA test. HBV infection status was determined by positive Hepatitis B surface antigen (HBsAg) or e-antigen (HBeAg), or HBV DNA test.

### Cancer outcomes

A total of 32 incident virus-associated cancers with etiologies linked to HPV, EBV, HHV-8, HBV, or HCV diagnosed during the study period were identified using International Classification of Diseases for Oncology, third edition (ICD-O-3) codes. Incident cancers were ascertained continuously during follow-up using cancer registry linkage data, available medical records and death certificates, and self-reported cancer diagnoses [20]. After cancers were classified based on anatomical site and histology [20], analyses focused on a composite category, “virus-associated cancers,” comprised of diagnoses associated with infection by HPV [anal cancer and head and neck squamous cell carcinomas (HNSCC)], EBV [non-Hodgkin lymphoma (NHL) and Hodgkin lymphoma (HL)], HHV-8 (Kaposi sarcoma [KS]), and HBV/HCV (liver cancer). Despite an established oncogenic role of HPV in development of skin SCC [23], skin cancers were excluded from the analysis given potential confounding effects of ultra-violet exposure, which was not ascertained. ICD-O-3 site codes for anal cancers were 21.0–21.8 and 19.9–20.9 based on a prior study in the MACS cohort [21]. ICD-O-3 site codes for HNSCC cases were 2.9, 4.9, and 10.2. The NHL cases were B-cell lymphomas (four diffuse large B-cell lymphomas, two follicular lymphomas, two malignant lymphomas of skin and unknown site, one mantle cell lymphoma, one chronic lymphocytic leukemia).

### Statistical analysis

Univariate or bivariate tests were conducted using Wilcoxon Rank-Sum test, paired *t* test, Pearson Chi-Square, or analysis of variance (ANOVA) for cohort characteristics by cancer diagnosis and CD4 count nadir. Exploratory analyses of longitudinal data utilized plots of mean values of immunological parameters for cases and controls during last 10 years of follow-up. Linear mixed-effects models were fit for longitudinal CD4 and WBC count as continuous dependent variables over the last 6 years of follow-up to determine the association between incident viral cancer diagnoses with these parameters and their rates of change. Observation time was left-truncated at 6 years prior to diagnosis because 428 participants (81.8%) had at least 12 semi-annual visits with immunological data, while data coverage became sparser going back further in time (Supplemental Material 1). Log2 transformation was applied to WBC values to improve normality of distributions. Models were adjusted for baseline age (at 6 years prior to follow-up), race, heavy smoking, FIB-4 > 1.45, and time to diagnosis; all models included random intercepts and slopes. Cox regression models were used to calculate adjusted hazard ratios (HR) of incident virus-associated cancer diagnoses associated with time-varying 2-year lagged CD4 cell count and CD4:CD8 ratio, CD4 count-nadir differential, and CD4 count and CD4:CD8 ratio nadirs during follow-up. We used a stepwise approach to evaluate Cox regression models, screening for predictors among these immunological parameters to evaluate their associations with risk. Modeling started with age, race, smoking, and CD4 count, followed by sequentially replacing the CD4 count variable with CD4 nadir, CD4:CD8 ratio, or CD4:CD8 ratio nadir. We hypothesized that including both CD4 count and CD4 count minus CD4 nadir in the model would provide a more dynamic representation of the relationship between low CD4 counts and cancer risk and therefore we extended models 1 and 2 by including both CD4 count minus CD4 nadir among the parameters tested. Models were adjusted for cumulative heavy smoking (≥ 0.5 packs/day average in last 6 years of follow-up) and race; age was used as the time scale. All analyses were performed using R version 3.2.

## Results

### Cohort characteristics

The study population of 32 virus-associated cancer cases and 500 matched controls had a median (IQR) age and follow-up of 35 (30–42) and 21 (8–26) years, respectively (Table 1). The majority of incident cancers were anal cancers and NHL (*n* = 9 cases each) followed by liver cancer (*n* = 6 cases), while incident HL, HNSCC, and KS accounted for three cases or less (Supplemental Material 2). Virus-associated cancers comprised the first diagnosis among two of four subjects with multiple cancer diagnoses (Supplemental Material 3); in the remaining two cases, virus-associated cancers were the second primary malignancy, diagnosed 17 and 2 years after previous diagnoses of thyroid cancer and prostate cancer, respectively. Compared to controls, virus-associated cancer cases were slightly older at entry, with shorter follow-up and higher percentage with HBV infection (*p* ≤ 0.003). Cases were similar to controls with regard to race, smoking, calendar period, HCV infection, number of sexual partners, and sexually transmitted infections (Table 1). Cases had a non-significant trend toward lower median CD4 cell counts compared to controls (858 vs. 947 cells/µl, respectively; *p* = 0.15), while CD4 nadir was significantly lower (512 vs. 620 cells/µl; *p* = 0.007) and percentage with CD4 nadir < 500 cells/µl was higher (46.9% vs. 27.4%; *p* = 0.03). There was a trend toward lower median CD4:CD8 ratio nadir in cases compared to controls (1.62 vs. 1.85, respectively; *p* = 0.08), while the percentage with CD4:CD8 ratio nadir < 1 was higher (56.2% vs. 29.2%; *p* = 0.003). Median time from CD4, CD4:CD8, CD8, and WBC nadir to cancer diagnosis ranged from 7.25 to 9.75 years.

### Immunological parameters prior to cancer diagnosis

**Fig. 1:**
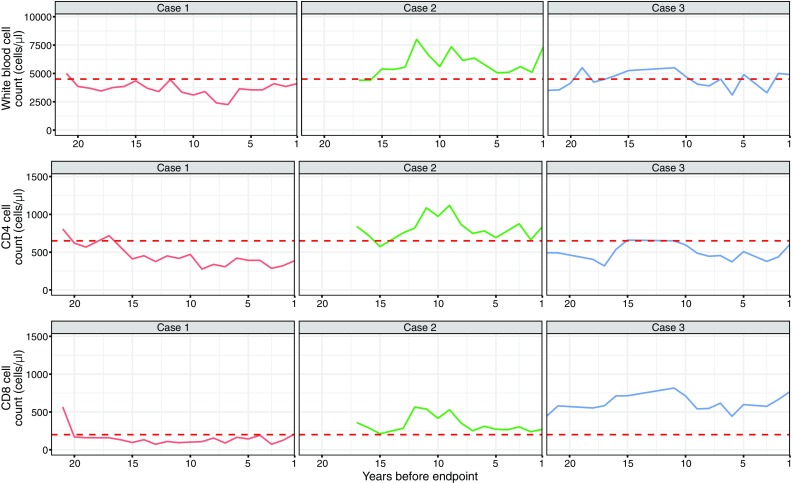
Longitudinal trends of immune parameters in three representative virus-associated cancer cases with immunological lab data available over 15 years or longer. Liver cancer, anal cancer, and Kaposi sarcoma were diagnosed in cases 1, 2, and 3, respectively. Dashed lines indicate lower reference range thresholds for normal values (4,500, 650, and 200 cells/µl for WBC, CD4, and CD8 counts, respectively)

**Fig. 2:**
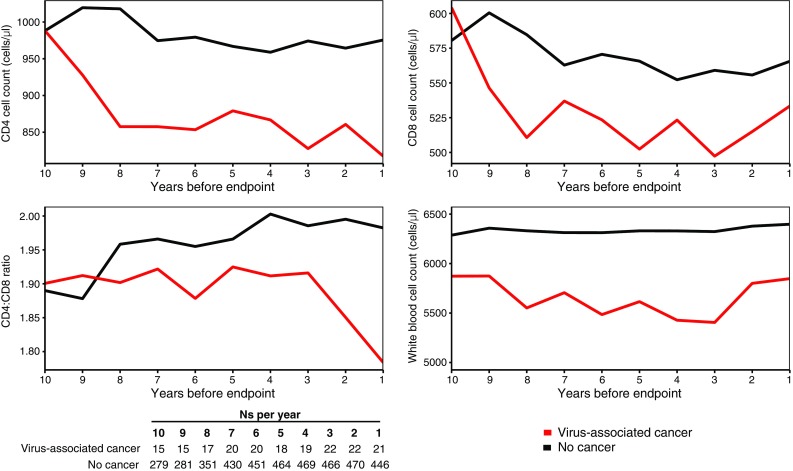
Mean longitudinal trajectories of immune parameters in groups by virus-associated cancer diagnosis. One subject was censored due to chronic use of antiviral medication for more than 10 years

**Fig. 3:**
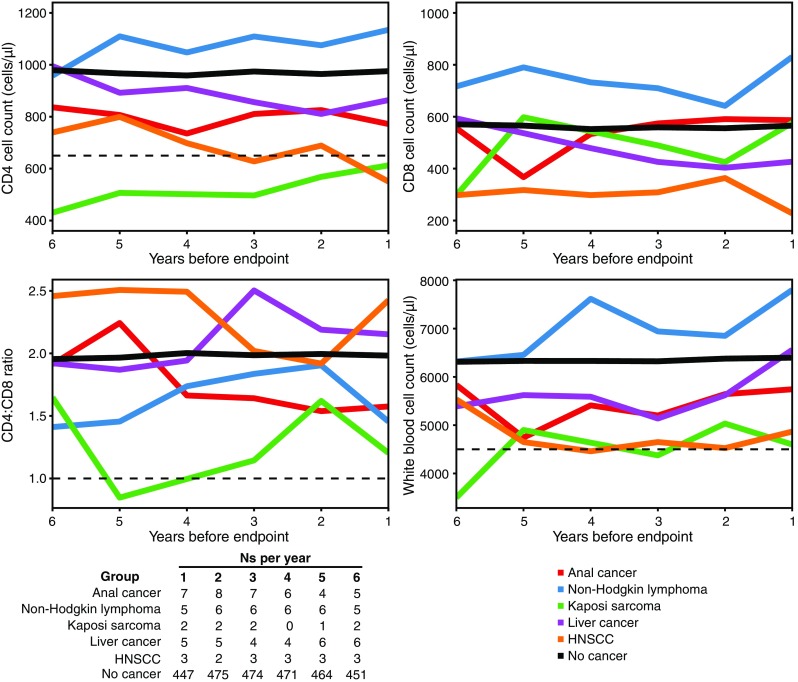
Mean longitudinal trajectories of immune parameters in groups by type of virus-associated cancer. Hodgkin lymphoma cases (*n* = 3) were excluded due to limited data. Dashed lines indicate lower reference range thresholds for normal values (4,500, 650, and 200 cells/µl for WBC, CD4, and CD8 counts, respectively)

Immunological parameters were first examined in exploratory analyses of individual and mean trajectories in cases and controls during follow-up, which indicated an optimal window between 6 and 10 years prior to diagnosis for evaluation of prognostic markers based on group differences and data coverage (Supplemental Material 1 and Figs. 1, 2, 3). Longitudinal trends of CD4, CD8, and WBC counts in individual virus-associated cancer cases showed variable patterns over 10 years or more prior to diagnosis, including chronically low counts for all three parameters, chronically low CD4 and WBC counts only, and declining CD4 and WBC counts; representative cases with immunological lab data available over 15 years or longer are shown in Fig. 1. The distribution of number of visits with immunological lab data for all cases and controls in the study cohort showed that 100%, 81.8%, and 63% of participants had immunological lab data for at least 2, 12, and 20 visits, respectively, corresponding to 1, 6, and 10 years of follow-up (Supplemental Material 1). Given this distribution of data coverage, exploratory analyses evaluated prognostic markers over 10 years for groups by cancer status and 6 years for groups by individual diagnoses. These exploratory analyses showed lower mean trajectories for immunological parameters, including CD4, CD8, WBC counts, and CD4:CD8 ratios, in cases compared to controls over a 10 year window (Fig. 2). When examined by individual diagnoses, subjects who developed NHL had slightly higher mean CD4, CD8, and WBC trajectories compared to those who developed solid-tissue tumors and controls (Fig. 3).

### Clinical characteristics associated with low CD4 counts

Next, we sought to examine clinical characteristics associated with low CD4 nadir in the study cohort. Although CD4 lymphopenia is often idiopathic, previous studies showed that medical conditions associated with CD4 lymphopenia in HIV-negative individuals include cirrhosis and other advanced liver disease [16–18]. Therefore, we examined liver disease-related factors in groups by CD4 nadir < 500 cells/µl (Table 2). The frequencies of HCV and HBV infection, heavy alcohol consumption, liver conditions, and cirrhosis did not differ between groups by CD4 nadir. However, HBV infection and cirrhosis were more common among cases compared to controls, regardless of CD4 nadir status. The proportion with FIB-4 scores > 1.45, a common threshold indicating liver fibrosis in HCV infection [22], was nearly twice as high in groups with CD4 count nadir < 500 cells/µl compared to those with CD4 nadir ≥ 500 cells/µl among both cases and controls (62.5% vs. 37.5%, and 34.4% vs. 20.3%, respectively). Platelet counts below the normal adult reference range (< 150 × 10^9^/l), a common finding in patients with advanced liver disease, were also more common in cases and controls with CD4 nadir < 500 cells/µl compared to corresponding groups with CD4 nadir ≥ 500 cells/µl (53.3% vs. 17.6%, *p* = 0.001, and 32.8% vs. 17.9%, *p* = 0.08, respectively).

**Table 2:** Immunological and liver disease parameters by CD4 nadir and virus-associated cancer status

	Controls (*n* = 500)			Cases (*n* = 32)		
	CD4 nadir ≥ 500 cells/µl (*n* = 363)	CD4 nadir < 500 cells/µl (*n* = 137)	*p*	CD4 nadir ≥ 500 cells/µl (*n* = 17)	CD4 nadir < 500 cells/µl (*n* = 15)	*p*
Hepatitis C (HCV) infection^a^	33 (9.1)	11 (8.0)	0.84	1 (5.9)	0 (0.0)	1.00
Hepatitis B (HBV) infection^a^	8 (2.2)	7 (5.1)	0.16	3 (17.6)	4 (26.7)	0.85
HBV core antibody positive^a^	177 (48.8)	68 (49.6)	0.94	8 (47.1)	11 (73.3)	0.25
Heavy alcohol use^b^	88 (24.8)	32 (24.4)	1.00	5 (31.2)	4 (26.7)	1.00
Liver conditions^a^	23 (6.3)	9 (6.6)	1.00	1 (5.9)	3 (20.0)	0.50
Liver cirrhosis^a^	3 (0.8)	2 (1.5)	0.89	1 (5.9)	2 (13.3)	0.90
Fibrosis-4 (FIB-4) score > 1.45^c^	72 (20.3)	44 (34.4)	**0.002**	3 (37.5)	5 (62.5)	0.61
FIB-4 score^c^, median (IQR)	0.96 (0.76, 1.21)	1.11 (0.87, 1.47)	< 0.001	1.28 (0.99, 2.82)	1.42 (1.22, 2.05)	0.67
Platelet count < 150 (× 10^9^/l)^a^	65 (17.9)	45 (32.8)	**0.001**	3 (17.6)	8 (53.3)	0.08
WBC nadir < 4,000 (cells/µl)^a^	73 (20.1)	68 (49.6)	< **0.001**	3 (17.6)	9 (60.0)	**0.035**
B-cell count nadir (cells/µl)^a^, mean (sd)	361.4 (145.26)	258.7 (108.96)	< **0.001**	385.0 (128.89)	241.3 (80.67)	**0.001**
Median B-cell count (cells/µl)^c^, mean (sd)	569.2 (193.27)	484.5 (164.20)	< **0.001**	628.8 (204.31)	432.3 (145.61)	**0.004**
T-cell count nadir (cells/µl)^a^, mean (sd)	1,124 (264)	667 (219)	< **0.001**	1,185 (427)	712 (268)	**0.001**
Median T-cell count (cells/µl)^c^, mean (sd)	1,634 (378)	1,290 (355)	< **0.001**	1,671 (480)	1,219 (356)	**0.006**
Visits with antibiotics use^a^, median (IQR)	5.00 (2.00, 11.00)	8.00 (3.00, 14.00)	**0.003**	3.00 (1.00, 7.00)	8.00 (3.50, 13.00)	**0.03**
Antibiotic use at ≥ 4 visits^a^	211 (58.1)	98 (71.5)	**0.008**	7 (41.2)	11 (73.3)	0.14

Data are *n* (%) unless otherwise indicated

Bold values indicate *p* < 0.05

^a^Anytime following enrollment to study endpoint

^b^≥ 14 drinks/week, or ≥ 5 drinks on one occasion at least monthly

^c^Nearest available value 1 year prior to endpoint

Next, we sought to determine if additional immunological parameters that could influence virus-associated cancer risk were detected among subjects with low CD4 nadir. To address this question, we evaluated total T- and B-cell and white blood cell counts in groups by CD4 nadir < 500 cells/µl. Total B-cell and T-cell count nadir and median summaries were determined for each subject, and summarized by cancer diagnosis (Table 2). Total B-cell count nadir and median were lower among cases and controls with CD4 nadir < 500 cells/µl compared to those with CD4 nadir ≥ 500 cells/µl (*p* = 0.004 and 0.02 for cases; *p* < 0.001 and *p* < 0.001 for controls, respectively). Trends for WBC and total T-cell counts paralleled those of CD4 cell counts; controls with CD4 nadir < 500 cells/µl had more than twice the percentage of subjects with WBC nadir < 4,000 cells/µl, and nearly twofold lower mean T-cell count nadir, compared to corresponding groups with CD4 nadir ≥ 500 cells/µl (*p* = 0.002).

Given that prolonged antibiotic use has been linked to alterations in immune defenses [24–26], we examined self-reported duration of antibiotic use during follow-up. Antibiotic use was reported at more visits in cases and controls with CD4 nadir < 500 cells/µl compared to those with CD4 nadir ≥ 500 cells/µl among cases and controls (median 5 vs. 8 visits, *p* = 0.003, and 3 vs. 8 visits, *p* = 0.03, respectively). However, we did not observe a trend of antibiotic use prior to CD4 nadir.

### Mixed-effects models

**Fig. 4:**
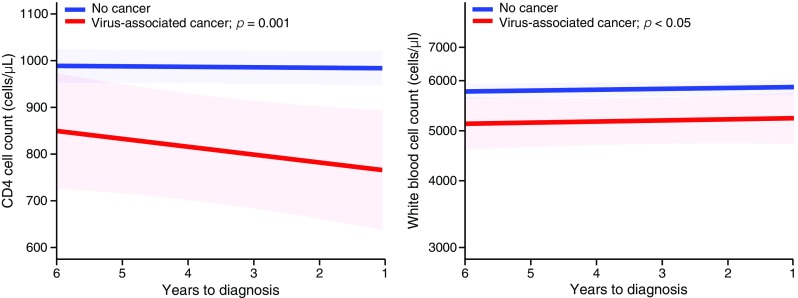
Mean longitudinal trajectories of CD4 and white blood cell counts in groups via mixed-effect models for virus-associated cancer vs. no cancer. Given discordant patterns for CD4 and WBC counts in NHL cases compared to other virus-associated cancers (see Fig. 3), NHL cases were excluded from these models. Full models adjusted for age, race, smoking, FIB-4 score, and time to diagnosis are shown in Supplemental Material 4

Given differences in CD4 and WBC counts in Figs. 2 and 3, we investigated the association of CD4 and WBC counts with incident virus-associated cancers within a 6-year time window in mixed-effects models. An incident diagnosis of virus-associated cancer was associated with lower CD4 cell and white blood cell counts in models adjusted for age, race, heavy smoking, and FIB-4 scores > 1.45 (*p* = 0.001 and 0.032, respectively; Fig. 4 and Supplemental Material 4). High FIB-4 was independently associated with lower CD4 and white blood cell counts, while heavy smoking was associated with higher counts (Supplemental Material 4).

### Cox models

To examine the prognostic value of CD4 and CD8 T-cell parameters relative to incident virus-associated cancers, Cox regression models were fit to CD4 cell count, CD4 cell count-nadir differential, and CD4:CD8 ratio and nadir with adjustments for age (as the time scale), race, and heavy smoking (Table 3). Lower CD4 cell count nadir showed a strong association with risk of incident virus-associated cancers (adjusted HRs/100 cells/µl decrease [95% CI] 1.31 [1.13, 1.51]; *p* < 0.001). Current CD4 cell count-nadir differential was a better predictor of incident virus-associated cancers (adjusted HRs/100 cells/µl decrease and 95% CI 1.27 [1.09, 1.48], *p* = 0.002) than recent CD4 count (adjusted HRs/100 cells/µl decrease [95% CI] 1.17 [1.03–1.33]; *p* = 0.014). Current CD4:CD8 ratios were not associated with increased risk of virus-associated cancers (*p* = 0.179); however, nadir CD4:CD8 ratio was associated with a three to fourfold increased risk of virus-associated cancers (adjusted HRs, 0.1 unit decrease and 95% CI 1.18 [1.06, 1.31]; *p* = 0.002). Heavy smoking was associated with a three to fourfold increased risk of virus-associated cancers, while race had no significant associations in these models (Table 3).

**Table 3:** Association of CD4 cell count or CD4:CD8 ratio and nadirs with virus-associated cancer diagnosis

	Virus-associated cancer^a^	
	HR (95% Cl)	*p*
Model 1		
CD4 count (per 100 cells/µl decrease)	1.17 (1.03, 1.33)	**0.014**
Heavy smoking^b^	4.54 (1.82, 11.29)	**0.001**
Black race	1.08 (0.38, 3.09)	0.881
Model 2		
CD4 nadir (per 100 cells/µl decrease)	1.31 (1.13, 1.51)	< **0.001**
Heavy smoking^b^	4.05 (1.65, 9.95)	**0.002**
Black race	1.34 (0.47, 3.82)	0.579
Model 3		
CD4 count (per 100 cells/µl decrease)	1.36 (1.15, 1.62)	< **0.001**
CD4 count minus CD4 nadir (per 100 cells/µl decrease)	1.27 (1.09, 1.48)	**0.002**
Heavy smoking^b^	4.79 (1.82, 12.59)	**0.002**
Black race	1.22 (0.42, 3.55)	0.709
Model 4		
CD4:CD8 ratio (per 0.1 unit decrease)	1.04 (0.98, 1.1)	0.179
Heavy smoking^b^	3.23 (1.35, 7.74)	**0.009**
Black race	1.17 (0.39, 3.48)	0.778
Model 5		
CD4:CD8 ratio nadir (per 0.1 unit decrease)	1.18 (1.06, 1.31)	**0.002**
Heavy smoking^a^	4.08 (1.55, 10.73)	**0.004**
Black race	1.19 (0.36, 3.93)	0.777

Multivariate Cox proportional hazards models adjusted for age

Bold values indicate *p* < 0.05

^a^Composite measure of first virus-associated cancer diagnosis of Kaposi sarcoma, non-Hodgkin lymphoma, Hodgkin lymphoma, anal cancer, head and neck squamous cell carcinoma, or liver cancer (*n* = 32 cases)

^b^0.5 packs/day or more on average over last 6 years of follow-up

## Discussion

In this longitudinal case–control study of HIV-uninfected men with median 21 years of follow-up, low CD4 T-cell count and nadir and low CD4:CD8 ratio nadir were associated with increased risk of virus-associated cancers (*p* ≤ 0.002 in Cox hazard models). Our data suggest these prognostic markers have predictive value for risk of virus-associated cancers over a 6- to 8-year window prior to diagnosis; a subset of cases had chronically low CD4 and/or low CD8 counts as long as 10 years or longer prior to diagnosis (Fig. 1). In Cox hazard models, low CD4 cell count and nadir were associated with increased hazard of virus-associated cancers (adjusted HRs [95% CIs] 1.17 [1.03–1.33], and 1.3 [1.13–1.5], respectively). Low CD4 count-nadir differential (adjusted HR [95% CI] 1.27 [1.09–1.48]) was a stronger predictor of incident virus-associated cancers than recent CD4 count. Low CD4:CD8 ratio nadir was also associated with increased hazard of virus-associated cancer (adjusted HR [95% CI] 1.18 [1.06–1.31]), while recent CD4:CD8 ratio did not show a significant association. The role of CD4 and CD8 T cells in immune control of viral infections is well established. CD4 cells are necessary for maintenance of CD8 cell functions [27], while depletion of CD4 T cells leads to CD8 T-cell exhaustion. Low CD4:CD8 ratios correlate with immune activation in virally suppressed HIV-infected individuals, and have prognostic significance for AIDS-related morbidity including some viral cancers [28]. Together, these findings underscore the importance of CD4 T cells in natural immune protection against viral cancers in healthy HIV-seronegative people.

Low CD4 cell counts could not be attributed to specific medical conditions, nor use of immunosuppressive drugs, in most virus-associated cancer cases in the study cohort. Twenty-nine subjects (4 cases and 25 controls) met criteria for idiopathic CD4 lymphopenia, defined by CD4 cell count < 300 for at least one visit. One liver cancer case was lymphophenic at multiple visits, with CD4 and CD8 cell counts < 300 and 100, respectively (Fig. 1, left panels); these observations together with our finding that FIB-4 > 1.45 is more frequent among subjects with CD4 nadir < 500 compared to those with CD4 nadir ≥ 500 are consistent with other studies linking lymphopenias with advanced liver disease [16, 18]. Lower platelet counts in groups with CD4 nadir < 500 may also reflect liver disease in some subjects. Antibiotic use was more frequent among cases and controls with CD4 nadir < 500 compared to subjects that maintained CD4 nadir ≥ 500. The significance of this finding remains unclear, but it could reflect increased susceptibility to bacterial infections in the setting of low CD4 counts or microbiome changes that influence immune function [24, 26, 29].

Distinct immunological profiles and trends were observed for subjects that developed NHL compared to those that developed solid-tissue cancers (Fig. 3), which may reflect differences in the natural history of EBV infection and EBV-related cancer compared to other oncogenic virus infections. Greater than 90% of the general population is exposed to EBV infection by their mid-twenties [4]; in addition to immunosuppression, host co-factors, oncogenic hits, and virus re-activation from latency play a role in development of EBV-related cancers [2]. The relative increase in mean CD4 and CD8 T-cell count trajectories prior to diagnosis in NHL cases compared to controls (Fig. 3) may reflect lymphocytosis driven by EBV-induced B-cell proliferation [30]. However, previous studies suggest that B-cell activation markers (e.g., sCD27, sCD30) and immunodeficiency markers (e.g., low CD4 and low CD4:CD8 ratio) represent different classes of early detection markers, linked to NHL through distinct mechanisms [8, 31].

The study cohort had a high prevalence (52.8%) of participants with positive tests for Hepatitis B core protein antibodies (anti-HBc), an indicator of past HBV infection, consistent with studies demonstrating higher rates of HBV in MSM compared to the general population [32]. All subjects with liver cancer had evidence of past or current HBV or HCV infection. Prior studies demonstrated lower CD4 cell counts in HIV-infected individuals positive for anti-HBc compared to HBc seronegatives [33]; in our cohort, the percentage of cases positive for anti-HBc among those with CD4 nadir < 500 cells/µl was higher than that of cases with CD4 nadir ≥ 500 cells/µl (73.3% vs. 47.1%), raising the possibility that prior HBV infection may impact immune functions that protect against some non-hepatic viral cancers [16–18]. Chronic HBV infection has been associated with T-cell exhaustion, but the association between anti-HBc positivity and T-cell exhaustion is unclear [34]. The impact of past or current HBV infection on risk of non-hepatic viral cancers is an open question that warrants further study.

To our knowledge, this is the first case–control study to examine immunological predictors of incident virus-associated cancer in a cohort of HIV-seronegative men. We acknowledge limitations of our study, however. The relatively small number of cancer outcomes and sample size limited adjustment for a large number of potential confounding factors through regression models and statistical power to detect some associations. Some cohort characteristics are more prevalent in MSM compared to the general population, including smoking, HBV/HCV infection, liver dysfunction, and sexually transmitted infections, which limits generalizability of our findings. For example, smoking has been associated with elevated risk of HNSCC and anal cancers after controlling for HPV-infection status and other factors, and could influence some findings [35–37]. Our analysis was limited by lack of data for other immune cell types, limiting our ability to evaluate findings within a broader immunological context. Although HIV serology testing was performed at semi-annual visits, three cancer cases lacked HIV serology testing within 4–5 years prior to cancer diagnosis so we cannot exclude the possibility that these individuals might have seroconverted between their last study visit and cancer diagnosis. We right-censored immunological data 1 year prior to cancer diagnosis and therefore cannot reach conclusions about immunological parameters at time of diagnosis. Lastly, serological testing and other lab data for EBV, HPV, and HHV-8 detection was not available, so we cannot reach any conclusions regarding infections with an oncogenic virus and development of virus-associated cancers.

In summary, our study shows that HIV-seronegative MSM with low nadir CD4 counts or low CD4:CD8 ratio nadirs have elevated risk of developing virus-associated cancers within a 6-year time window. Furthermore, our studies suggest that past or current HBV infection coupled with low CD4 count or nadir or low CD4:CD8 ratio is a profile associated with increased risk of developing virus-associated cancers in otherwise healthy HIV-seronegative men. Such individuals might be candidates to screen for treatable oncogenic viral infections or pre-malignant lesions to reduce their cancer risk.

## Electronic supplementary material

Below is the link to the electronic supplementary material.

Supplementary material 1 (PDF 71 KB)

Supplementary material 2 (PDF 77 KB)

Supplementary material 3 (PDF 68 KB)

Supplementary material 4 (PDF 79 KB)
