# Supplementary material for: Low T-cell subsets prior to development of virus-associated cancer in HIV-seronegative men who have sex with men
Source: Cancer Causes Control. 2018 Oct 12;29(11):1131–42. doi: 10.1007/s10552-018-1090-4 (PMC6245112; doi:10.1007/s10552-018-1090-4)

Supplemental Material 1. Distribution of number of visits with immunological data for subjects in the study cohort (n=532).

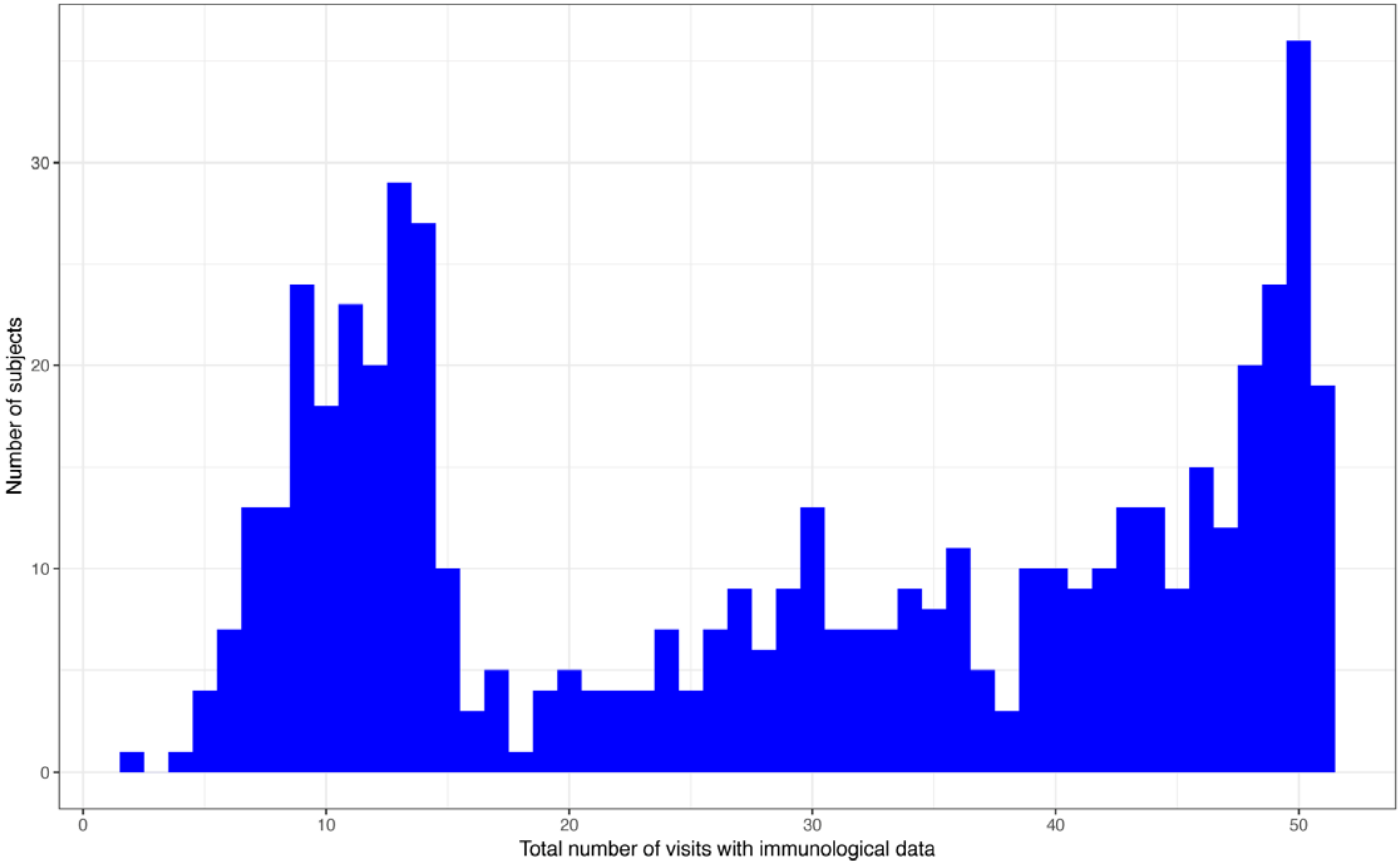

Supplement: Supplementary file 1 — Supplementary material 1 (PDF 71 KB) [file 10552_2018_1090_MOESM1_ESM.pdf]
