# Supplementary material for: Low T-cell subsets prior to development of virus-associated cancer in HIV-seronegative men who have sex with men
Source: Cancer Causes Control. 2018 Oct 12;29(11):1131–42. doi: 10.1007/s10552-018-1090-4 (PMC6245112; doi:10.1007/s10552-018-1090-4)
Supplement: Supplementary file 2 — Supplementary material 2 (PDF 77 KB) [file 10552_2018_1090_MOESM2_ESM.pdf]

Supplemental Material 2. Frequency of incident virus-associated cancers.

Cancer	Count
Anal cancer	9
Hodgkin lymphoma	3
Kaposi sarcoma	2
Liver cancer	6
Non-Hodgkin lymphoma	9
Head and neck squamous cell carcinoma	3
