# Supplementary material for: Low T-cell subsets prior to development of virus-associated cancer in HIV-seronegative men who have sex with men
Source: Cancer Causes Control. 2018 Oct 12;29(11):1131–42. doi: 10.1007/s10552-018-1090-4 (PMC6245112; doi:10.1007/s10552-018-1090-4)
Supplement: Supplementary file 3 — Supplementary material 3 (PDF 68 KB) [file 10552_2018_1090_MOESM3_ESM.pdf]

Supplemental Material 3. Subjects with multiple cancer diagnoses

Subject	Virus-associated cancer		Non-virus-associated cancer		
	First diagnosis	Second diagnosis	First diagnosis	Second diagnosis	Time interval between diagnoses
1		Hodgkin lymphoma	Thyroid cancer		17 years
2		Anal cancer	Prostate cancer		2 years
3	Non Hodgkin lymphoma			Prostate cancer	9 years
4	Kaposi sarcoma			Prostate cancer	14 years
