# Supplementary material for: Low T-cell subsets prior to development of virus-associated cancer in HIV-seronegative men who have sex with men
Source: Cancer Causes Control. 2018 Oct 12;29(11):1131–42. doi: 10.1007/s10552-018-1090-4 (PMC6245112; doi:10.1007/s10552-018-1090-4)
Supplement: Supplementary file 4 — Supplementary material 4 (PDF 79 KB) [file 10552_2018_1090_MOESM4_ESM.pdf]

Supplemental Material 4. Longitudinal trends of CD4 and white blood cell counts proximal to virus-associated cancer diagnosis from mixed-effects models.

	CD4 cell count (cells/µl)			White blood cell count (cells/µl) (log_2)		
	**Estimate**	**SE**	**p-value**	**Estimate**	**SE**	**p-value**
Virus-associated cancer	-233.667	71.748	**0.001**	-0.185	0.086	**0.032**
Heavy smoking[a]	179.515	28.533	**<0.001**	0.245	0.035	**<0.001**
Age proximal to diagnosis[b]	1.590	1.138	0.163	0.000	0.000	**0.041**
Black race	-7.809	28.443	0.784	0.002	0.001	0.201
FIB-4 > 1.45[c]	-122.874	29.688	**<0.001**	-0.145	0.035	**<0.001**
Time to diagnosis[d]	-0.084	0.164	0.608	-0.196	0.037	**<0.001**

Heterogeneity was assumed between groups by cancer diagnosis; therefore, group by time interaction was included in the models despite non-significant estimates. Given discordant patterns for CD4 and WBC counts in NHL cases compared to other virus-associated cancers (see Figure 3), NHL cases were excluded from these models.

[a]Smoking 0.5 packs per day or more on average during follow-up

[b]Age 6 years prior to endpoint.

[c]Time-updated.

[d]From 6 years prior to endpoint
